# Medical News

**Published:** 1855-01

**Authors:** 


### MEDICAL NEWS.

Hullihen's Ear Syringe.—We have already more than once invited attention to this admirably simple and efficient little instrument. We do so again for the purpose of apprising our medical friends that it may be obtained at the pharmaceutical establishment of Mr. Henry C. Blair, of this city, who has undertaken to furnish it in the most approved form, and has had it very conveniently put up in a neat cylindrical box, for being carried about and shielded from the dust.

We can strongly recommend this instrument to both practitioners and invalids as a most desirable substitute for the ordinary syringe and basin. Even as a self-injector, in the hands of an intelligent patient, under the direction of a medical attendant, it must be regarded as an invaluable saver of trouble and annoyance to both parties, and should be considered as indispensable to each individual requiring such manipulation for his ears as a special tooth-brush is for the daily cleansing of his teeth. There is another virtue in Dr. Hullihen's invention, which, in these trading days of multiple adjusters, supporters, trusses, et id omne genus, entitles it to signal admiration and encouragement:—it is not encumbered with a patent.

---

AMERICAN MEDICAL ASSOCIATION.—The eighth Annual Meeting of the American Medical Association will be held in the city of Philadelphia, on Tuesday, May 1, 1855.

The Secretaries of all Societies and other bodies entitled to representation in the Association, are requested to forward to the undersigned correct lists of their respective delegations *as soon as they may be appointed;* and it is *earnestly* desired by the Committee of Arrangements that the appointments be made at as early a period as possible.

The following are extracts from Article 2d of the Constitution:—

"Each local Society shall have the privilege of sending one delegate for every ten of its regular resident members, and one for every additional fraction of more than half of this number. The faculty of every regularly constituted medical college, or chartered school of medicine, shall have the privilege of sending two delegates. The professional staff of every chartered or municipal hospital containing a hundred inmates or more, shall have the privilege of sending two delegates, and every other permanently organized medical institution of good standing shall have the privilege of sending one delegate.

"Delegates representing the medical staff of the United States army and navy shall be appointed by the Chiefs of Army and Navy Medical Bureaux.

"The number of delegates so appointed shall be four from the army medical officers, and an equal number from the navy medical officers."

The latter clause, in relation to delegates from the army and navy, was adopted as an amendment to the constitution at the meeting of the Association, held in New York, in May, 1853.

FRANCIS WEST, M.D.,
One of the Secretaries, 352 Chestnut st., Philadelphia.

The medical press of the United States is respectfully requested to copy the above.

Obituary.—The announcement of the death of Dr. Golding Bird, at the early age of thirty-nine years, will, we feel assured, excite a very general feeling of regret throughout the profession of this country. Dr. Bird was very highly appreciated here. His admirable work on "Urinary Deposits," which opened an almost entirely new field for research to the physician, attained at once a most brilliant success, and is still regarded as the highest authority upon the subject.

For some years previous to his death, Dr. Bird had suffered from disease of the heart; a short time before that event, he had an attack of hæmaturia, which soon "became associated with other and unerring evidence of renal calculus." This was followed by pyelitis, which ended his career on the 27th of October.

It is with much regret that we have also to announce the death of Prof. Edward Forbes, of the University of Edinburgh. This distinguished naturalist died on the 18th of November, in the 39th year of his age. It is not too much to say that science has not lost a nobler son during the present century; and there are none who enjoyed the advantages of his teaching or the honor of his friendship but will deeply feel the calamity which has befallen them. At a very early age, when a student in the University of Edinburgh, he gave strong evidences of talents of a very superior order. The Botanical Society of Edinburgh—one of the most flourishing scientific bodies in Great Britain—owes its origin to the late Professor and a few of his fellow students, more than one of whom were his late colleagues. After delivering a course of extra academical lectures in natural history he proceeded to the East, as naturalist to a scientific expedition. To this expedition are due not a few important contributions to science, and, we regret to say, to it we have to attribute his early death. Returning to London he was elected to the chair of Botany in King's College, which he filled with honor to himself and advantage to the college. The Geological Society acknowledged his high merit by electing him to every honorary office which it could confer; and, on the establishment of the School of Practical Geology and Designs, he was at the head of the Palæontological department. Last April, on the death of the venerable Jameson, Prof. Forbes, to the delight of all, had the appointment conferred on him; and our readers will remember with pleasure the extracts that we gave, in this journal, from his introductory lecture. In May he commenced the usual summer course of lectures. Though suffering from disease, he again opened his class on the 2d of November, but could only continue for six days. He died after nine days' illness, leaving a blank in the University which it will be difficult to fill up.

